# The Development of Immunological Assays to Evaluate the Level and Function of Antibodies Induced by Klebsiella pneumoniae O-Antigen Vaccines

**DOI:** 10.1128/msphere.00680-22

**Published:** 2023-03-06

**Authors:** Helen R. Wagstaffe, Marina Johnson, Guled Osman, Patricia Martin, Paula Carranza, David Goldblatt

**Affiliations:** a Great Ormond Street Institute of Child Health, University College London, London, UK; b LimmaTech Biologics AG, Schlieren, Switzerland; U.S. Food and Drug Administration

**Keywords:** *Klebsiella pneumoniae*, vaccine, immunogenicity, multiplex assay, opsonophagocytic killing assay, functional assay

## Abstract

Klebsiella pneumoniae, a Gram-negative bacterium, has been listed as a critical pathogen for urgent intervention by the World Health Organization. With no licensed vaccine and increasing resistance to antibiotics, Klebsiella pneumoniae causes a high incidence of hospital- and community-acquired infections. Recently, there has been progress in anti-Klebsiella pneumoniae vaccine development, which has highlighted the lack of standardized assays to measure vaccine immunogenicity. We have developed and optimized methods to measure antibody level and function after vaccination with an in-development Klebsiella pneumoniae O-antigen vaccine. We describe the qualification of a Luminex-based multiplex antibody binding assay and both an opsonophagocytic killing assay and serum bactericidal assay to measure antibody function. Serum from immunized animals were immunogenic and capable of binding to and killing specific Klebsiella serotypes. Cross-reactivity was observed but limited among serotypes sharing antigenic epitopes. In summary, these results demonstrate the standardization of assays that can be used to test new anti-Klebsiella pneumoniae vaccine candidates, which is important for moving them into clinical trials.

**IMPORTANCE** There is no licensed vaccine for the prevention of Klebsiella pneumoniae infections, and increasing levels of antibiotic resistance make this pathogen a high priority for vaccine and therapeutic development. Standardized assays for testing vaccine immunogenicity are paramount for the development of vaccines, and so in this study, we optimized and standardized both antibody-level and function assays for evaluating in-development K. pneumoniae bioconjugate vaccine response in rabbits.

## INTRODUCTION

Klebsiella pneumoniae is a Gram-negative bacterium belonging to the family of *Enterobacteriaceae*. It is opportunistic pathogen which can act as a commensal in the healthy flora of human mouth, skin, and intestine but also causes a high incidence of hospital- and community-acquired infections (1). K. pneumoniae commonly presents as urinary tract infections, pneumonia, soft tissue infections, or bacteremia. There is no licensed vaccine to prevent K. pneumoniae infection nor a candidate vaccine in clinical trials at this time.

K. pneumoniae bacteria are naturally resistant to antibiotics, such as aminopenicillins and carboxypenicillins, and in the past decades, this pathogen has developed several more strategies to resist different classes of antibiotics. Isolates of K. pneumoniae are carrying genes encoding extended-spectrum beta-lactamases (ESBLs) and mutations leading to fluoroquinolone resistance, and there has been an alarming rise of carbapenem-resistant and carbapenemase-producing K. pneumoniae isolates. The World Health Organization (WHO) considers carbapenem-resistant K. pneumoniae as a threat and therefore listed it among the “critical” priority pathogens for urgent intervention (2, 3).

The bacterial surface of K. pneumoniae displays two classes of immunogenic polysaccharides, namely, capsular K antigens and O antigens. The O antigens are more conserved with just 8 different O antigens identified versus the 77 K antigens; therefore, it is thought that vaccine development based on O antigens will allow broad coverage of many K. pneumoniae strains by including only a few O-antigen components (4). Epidemiology studies considering antibiotic resistances, population, country, and pathologies have shown that using 4 out of 8 lipopolysaccharide (LPS) serotypes (O1, O2a, O2afg, and O3b) can achieve a coverage of 60 to 99% of infections (4–11). Despite some progress in vaccine development over recent years, progression may have been hindered by the lack of fully standardized assays to measure immunity to K. pneumoniae, in particular anti-K. pneumoniae LPS antibody concentration and function.

The fully standardized Streptococcus pneumoniae opsonophagocytic killing assay (OPA) has been used for many years to measure the immunogenicity to pneumococcal conjugate vaccines (12). Both OPA and serum bactericidal assay (SBA) methods have been used to measure anti-K. pneumoniae antibody function in serum in the past (13, 14); however, variation in methodology and reagents and the lack of assay qualification mean these assays cannot be used for vaccine studies or clinical trials. Opsonophagocytosis and other mechanisms of antibody-induced killing have been shown to be important in protection against K. pneumoniae (15, 16). In this paper, we describe the development and optimization of functional antibody assays and multiplex binding assay that allow for the measurement of anti-K. pneumoniae immunity after vaccination.

## RESULTS

### Optimization and qualification of a multiplex assay to quantify anti-K. pneumoniae LPS antibodies.

**FIG 1:**
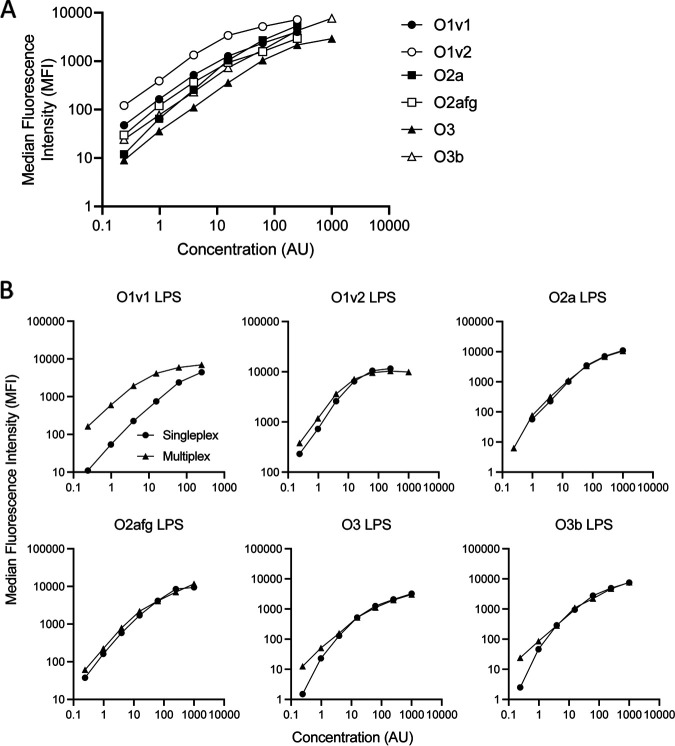
Klebsiella pneumoniae Luminex assay development. LPS conjugated beads in multiplex were incubated with a multiplex standard serum (a pool of sera from rabbits vaccinated with monovalent bioconjugate vaccines against O1v1, O1v2, O2a, O2afg, O3, and O3b K. pneumoniae serotypes), detection antibody was added and the MFI was determined (A), and the multivalent standard serum was incubated with single or multiplex bead preparations and the MFIs were compared (B).

In order to quantify the level of anti-LPS antibody in rabbit sera, a 6-plex serological Luminex assay was developed. Six carboxylated MagPlex microsphere sets were coupled to purified LPS of six different K. pneumoniae serotypes. A minimum of 3 LPS concentrations were tested for optimal bead coupling, and the R-phycoerythrin conjugated goat anti-rabbit IgG detection antibody was titrated for optimal concentration. Conjugation concentrations between 18 μg/mL and 8 μg/mL, with a detection antibody concentration of 3 μg/mL, were chosen due to maximal mean fluorescence intensities (MFIs) with positive serum and low background (blank) MFIs (<150 MFI) with assay buffer alone. Conjugated beads were tested with postvaccination monovalent and multivalent standard serum (a pool of postdose 3 immunization sera from each monovalent vaccinated rabbit), and Fig. 1A shows the standard curve generated for each serotype using the optimized 6-plex assay conditions.

To assess the impact of serotype cross-reactivity, the MFI of multivalent standard serum in the 6-plex assay was compared with that generated from an assay run with single-plex beads. The MFIs of multivalent standard serum run in 6-plex and single-plex were comparable for all LPS types except O1v1 where the 6-plex assay resulted in higher MFIs than with the single-plex assay (Fig. 1B).

The average coefficient of variation (CV) across all serotypes for intra-assay and interassay precision was 7.7% and 8.9%, respectively. The average percentage CV between replicates was 5.7%. The percentage recovery of the top standard curve dilution was 93 to 105%, and the average relative accuracy (the percentage CV between AU data obtained from three different dilutions of QC serum, run on three nonconsecutive days) was 7.2%. The linearity was between 0.990 and 0.998 (R^2^). See Table 1 for individual serotype data.

**TABLE 1:** Luminex assay qualification

Parameter	Data by serotype					
	O1v1	O1v2	O2a	O2afg	O3	O3b
Precision						
Intra-assay CV (%)	8.9	8.5	4.9	7.2	8.7	7.9
Interassay CV (%)	10.8	9.9	6.0	8.0	9.4	9.2
Replicates (CV [%])	3.9	5.1	5.6	6.0	6.4	7.1
Recovery (%)	100	105	100	102	97	93
Relative accuracy (CV [%])	8.4	4.8	7.6	4.9	7.9	9.6
Linearity (R^2^)	0.990	0.994	0.992	0.996	0.990	0.998
LLOD (MFI)	118.98	127.25	144.32	118.46	139.89	203.94
LLOQ						
MFI	475.94	509.02	577.28	473.83	559.57	815.75
concn (AU)	0.645	0.296	1.129	0.804	1.569	1.177

The reproducibility of the bead conjugation was tested by reconjugating a new set of beads using the same methodology and LPS concentrations >3 months after the first; beads were retested in the assay with the multivalent QC and multivalent standard serum. The MFIs generated were comparable for all LPS types between old and new bead conjugations (see Fig. S1A to F in the supplemental material).

10.1128/msphere.00680-22.1 FIG S1 Luminex bead conjugation reproducibility. The conjugation of each bead to PLL-modified Kp LPS was repeated with a new batch of beads (new), and the MFI generated from multiplex standard serum in a 6-plex assay on O1v1 LPS (a), O1v2 LPS (b), O2a LPS (c), O2afg LPS (d), O3LPS (e), and O3bL PS (f) conjugated beads was compared with the previously conjugated bead batch (old). Download FIG S1, PDF file, 0.1 MB. Copyright © 2023 Wagstaffe et al. 2023 Wagstaffe et al. https://creativecommons.org/licenses/by/4.0/ This content is distributed under the terms of the Creative Commons Attribution 4.0 International license.

### Luminex assay lower limits.

Luminex assay sensitivity was determined for each bead set, and the lower limit of detection (LLOD) was determined from 20 blank wells containing LPS-coupled beads and detection antibody in assay buffer alone. The lower limit of quantification (LLOQ) was determined as 4 times the LLOD, and interpolated concentration was shown in arbitrary units (AU). The LLOQ varied between serotypes, with a maximum of 1.569 (O3) and minimum of 0.296 (O1v2), and values obtained below the LLOQ of the serotype were assigned a value of half LLOQ for quantitative purposes (Table 1).

### Luminex assay cross-reactivity and specificity.

**FIG 2:**
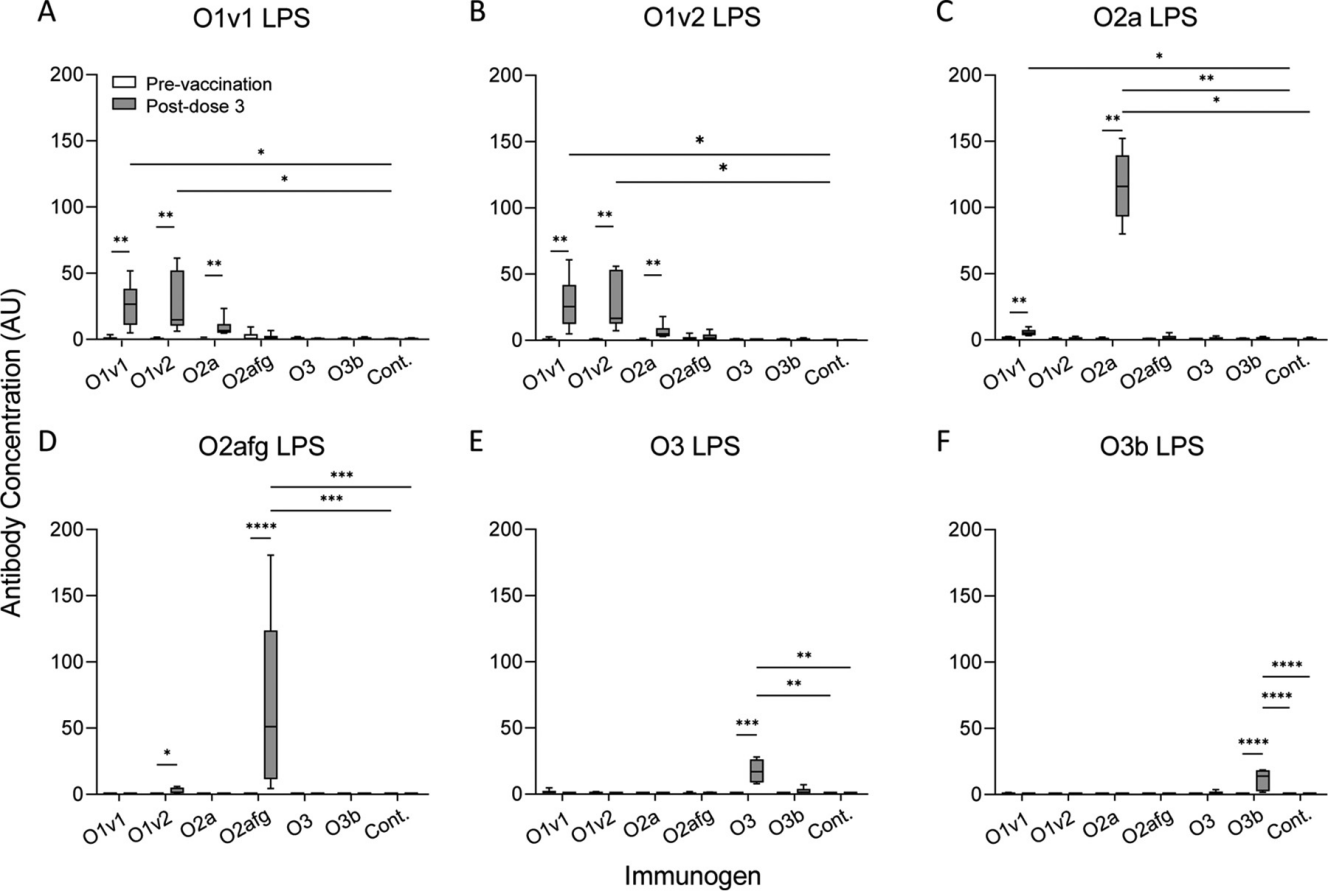
The concentration of anti-LPS antibody in rabbits vaccinated with monovalent Klebsiella pneumoniae bioconjugate vaccines. Rabbit sera from before (prevaccination) and after (postdose 3) vaccination with monovalent bioconjugate vaccines against each K. pneumoniae serotype (immunogen; *n* = 6 per group) or control (PBS) were run on the multiplex Luminex assay. The concentration of antibody against O1v1 LPS (A), O1v2 LPS (B), O2a LPS (C), O2afg LPS (D), O3 LPS (E), and O3b LPS (F) was determined in arbitrary units (AU) by interpolation from the standard curve. Comparisons between paired pre- and postvaccination time points were made using Wilcoxon signed-rank test and between vaccination groups using Kruskal-Wallis test with Dunn’s correction for multiple comparisons. *, *P < *0.05; **, *P < *0.01; ***, *P < *0.001; ****, *P < *0.0001. Cont. = control.

Anti-sera from individual rabbits vaccinated with monovalent bioconjugate vaccines against each of the six K. pneumoniae serotypes were assayed, and the concentration of each antibody was determined. In addition to homologous LPS, several antisera bound heterologous LPS (Fig. 2). Anti-O1v1 serum bound O1v2 and O2a LPS (Fig. 2A to C), anti-O1v2 serum bound O1v1 and O2afg LPS (Fig. 2A to D), and anti-O2a serum bound O1v1 and O1v2 LPS (Fig. 2A to C). Anti-O3 serum bound homologous O3 LPS but only weakly to heterologous O3b LPS and similarly visa versa (Fig. 2E and F).

To assess assay specificity, the multivalent standard serum was preincubated with each LPS to block homologous or heterologous binding, the 6-plex assay was run as normal, and percentage inhibition was calculated in relation to a zero-inhibition control containing serum preincubated with assay buffer alone (Table 2). Inhibition with homologous LPS was >98%; inhibition with heterologous, non-cross-reactive LPS was <11%; and inhibition of cross-reactive antibody binding was up to 97% (Table 2).

**TABLE 2:** Luminex specificity

Competing LPS	Percentage inhibition of binding by serotype^*a*^					
	O1v1	O1v2	O2a	O2afg	O3	O3b
O1v1	100	89	90	−3	11	1
O1v2	97	98	27	84	8	−6
O2a	12	3	99	6	8	1
O2afg	3	9	13	98	3	−2
O3	4	2	4	−9	98	22
O3b	−5	−6	4	−9	7	99

a Cells shaded gray represent homologous LPS.

### Development of a K. pneumoniae killing assay to measure anti-K. pneumoniae LPS antibody functionality in rabbit sera.

Initially, multiple K. pneumoniae strains were tested for suitability in an *in vitro* killing assay. Three strains, namely, O1v2 K136, O2a K18, and O2afg K140, were initially chosen due to their sensitivity to killing in response to immune rabbit serum and human intravenous immunoglobulin (IVIG) and relatively low nonspecific killing (NSK) (the killing induced by complement alone, in the absence of antisera).

**FIG 3:**
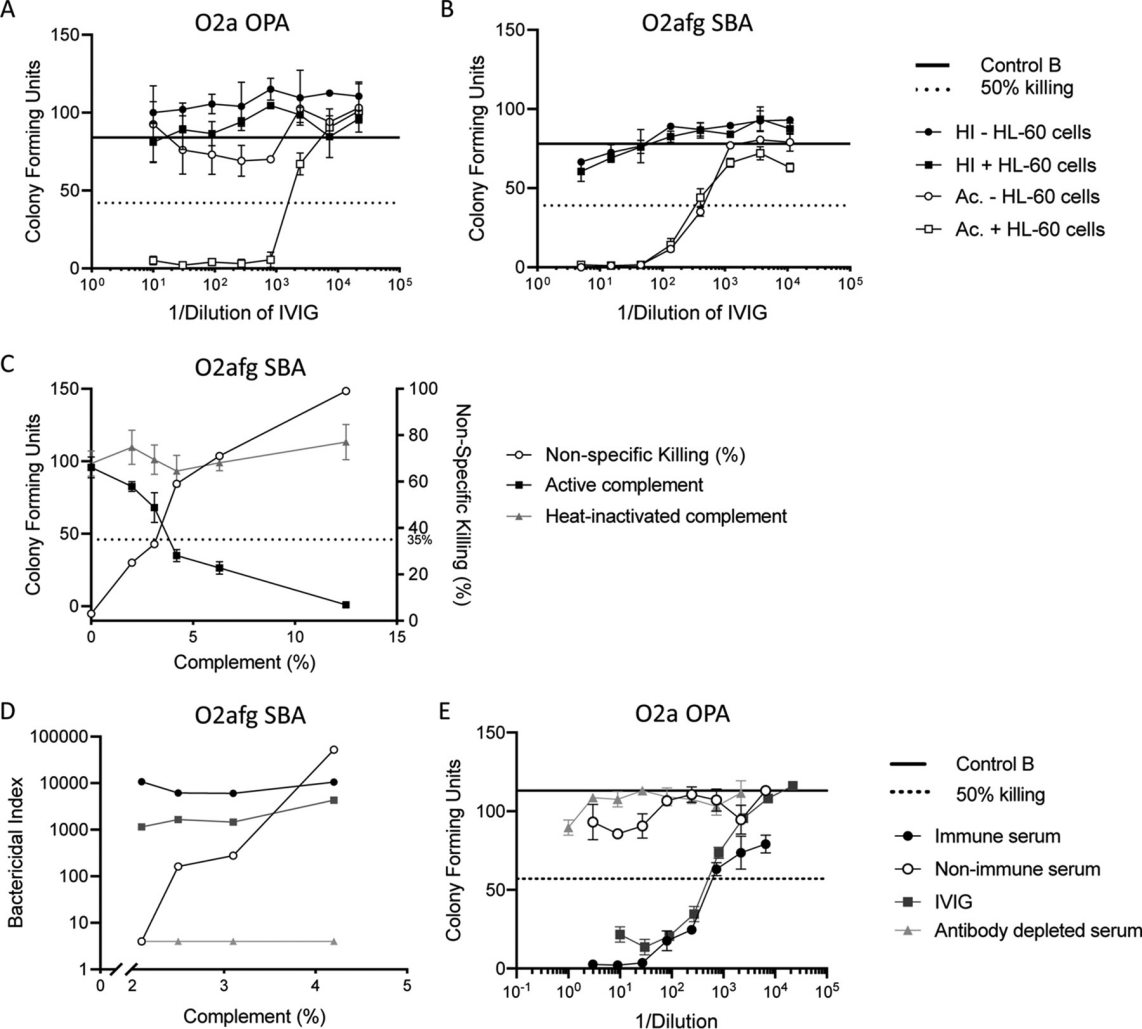
OPA and SBA optimization. Killing of O2a K18 (A) and O2afg K140 (B) was tested in response to IVIG in the presence and absence of HL-60 cells and with active (Ac.) or heat-inactive (HI) complement. A titration of active or HI complement in the O2afg SBA and NSK was assessed (C). A titration of complement with O2afg antiserum, prevaccination serum (nonimmune serum), IVIG, or antibody-depleted human serum and the bactericidal index (BI) were calculated (D). O2a antiserum, nonimmune serum, IVIG, and antibody-depleted serum were also tested on the O2a OPA (E). Graphs show mean CFU ± SD, bactericidal index, and percentage NSK.

IVIG was tested against the strains with active or heat-inactivated (HI) complement in the presence or absence of HL-60 cells for phagocytosis. As there was no reduction in CFU past 50% with HI complement or in the absence of HL-60 cells in the O2a OPA (Fig. 3A), killing O2a K18 by IVIG was dependent on HL-60 cells and the active complement. O1v2 OPA was similarly dependent on HL-60 cells and the active complement (data not shown). Whereas, killing of O2afg K140 was dependent on the active complement but independent of HL-60 cells as killing with active complement was similar with and without HL-60 cells (Fig. 3B). The O2afg killing assay was therefore further developed as an SBA, whereas O1v2 and O2a were developed as OPAs.

The O2afg K140 strain was more sensitive to the complement than O1v2 K136 and O2a K18; therefore, the concentration of the complement in the SBA was titrated to determine an optimal concentration for serum-induced killing but to keep a low percentage of NSK. Lowering the concentration of the complement resulted in reduced killing in the presence of active complement and decreased the percentage of NSK (Fig. 3C). The bactericidal index (BI) in response to immune rabbit serum and human IVIG was sustained with lower complement concentrations, and antibody-depleted serum remained negative at all concentrations. However, the BI of nonimmune rabbit serum (prevaccination) increased with increasing complement concentration (Fig. 3D). To meet an acceptability criterion of <35% NSK and to keep background levels of killing with prevaccination sera low, a concentration of 2% complement was chosen.

O2a K18 (and O1v2 K136, data not shown) was similarly killed in response to immune rabbit serum and human IVIG but not nonimmune (prevaccination) rabbit serum or antibody-depleted serum (Fig. 3E).

### OPA and SBA repeatability.

**FIG 4:**
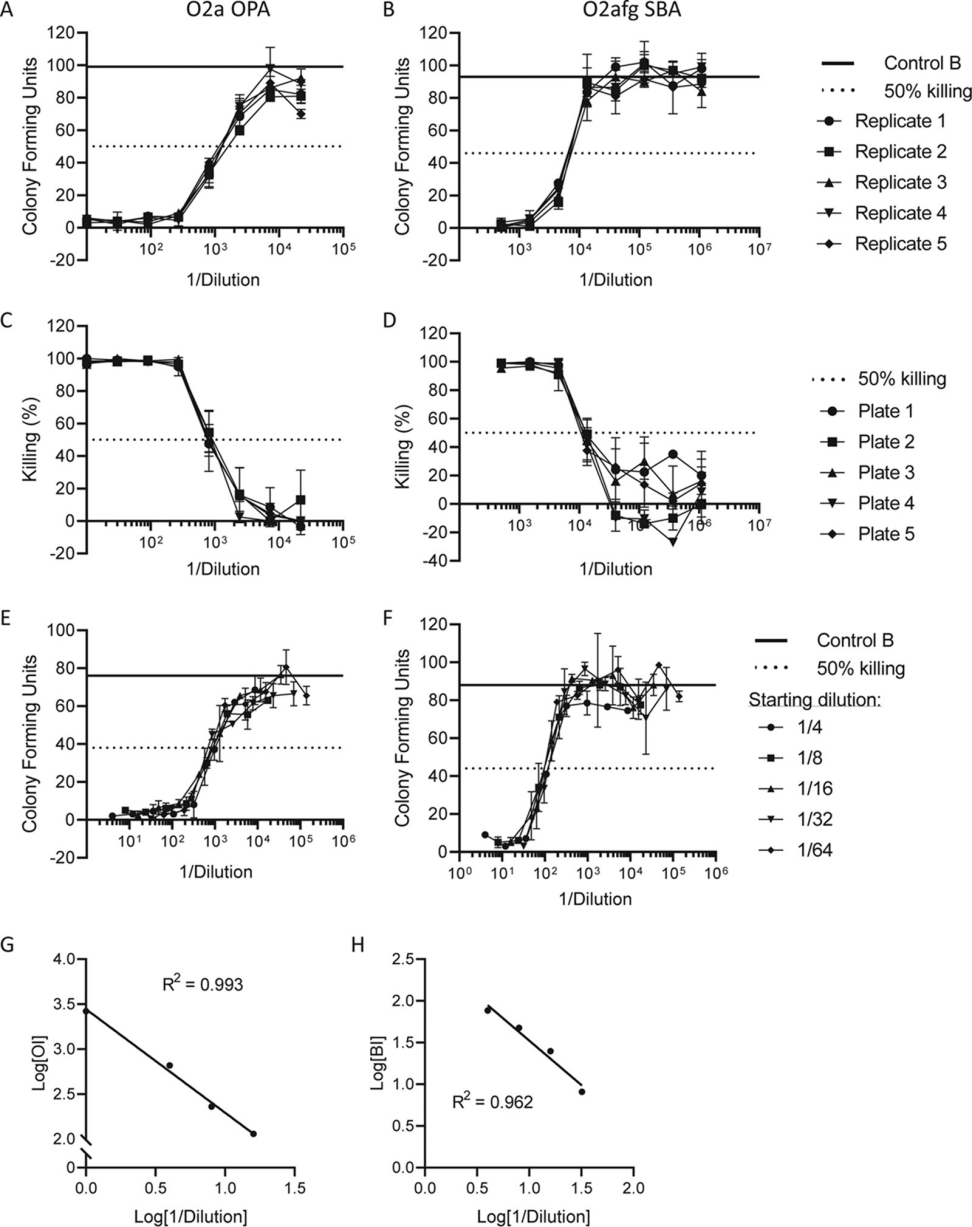
OPA and SBA qualification. The intra-assay (A, B), interassay (C, D), relative accuracy (E, F), and linearity (G, H) of O2a OPA (A, C, E, G) and O2afg SBA (B, D, F, H) were determined. Graphs show mean CFU ± SD or percentage killing calculated as 1 − CFU/control B × 100 from one representative experiment.

Assay precision was tested using one positive sample (monovalent immune rabbit serum or human IVIG) that was run 5 times on one plate (intra-assay) or once on 5 different plates (interassay) and repeated 3 times over 3 nonconsecutive days. Figure 4A and C (O2a OPA) and Fig. 4B and D (O2afg SBA) show representative data of the intra-assay and interassay repeatability, respectively. The overall intra-assay coefficient of variation between the resulting opsonic index (OI) or BI was 7.7%, 14.1%, and 4.1% for O1v2 OPA, O2a OPA, and O2afg SBA, respectively. The overall interassay coefficient of variation was 26.7%, 20.7%, and 16.0%, respectively. When variability was compared over 3 nonconsecutive days, >80% of OI/BI values obtained were within one 3-fold dilution of the mean (Table 3).

**TABLE 3:** OPA and SBA assay qualification

Parameter	Results by assay		
	O1v2 OPA	O2a OPA	O2afg SBA
Nonspecific killing (avg *n* = 10) (%)	16.7	14.6	27.1
Precision			
Intra-assay CV (%)	7.7	14.1	4.1
Interassay CV (%)	26.7	20.7	16
Relative accuracy (CV [%])	19.3	14	11.6
Linearity (R^2^)	0.885	0.993	0.962
LLOQ	11	8	12

The relative accuracy was measured by running the same positive sample at five different starting dilutions; the coefficient of variation between resulting OI/BI was 19.3%, 14.0%, and 11.6% (Fig. 4E and F; Table 3). Linearity was assessed by running antibody-depleted human serum spiked with four different concentrations of positive sample. The correlation between initial sample dilution (log transformed) and resultant OI/BI value (log transformed) was 0.885, 0.993, and 0.962 (R^2^) for O1v2 OPA, O2a OPA, and O2afg SBA, respectively (Fig. 4G and H; Table 3).

### OPA and SBA lower limit of quantification.

LLOQ was determined by spiking low concentrations of positive-control serum into antibody-depleted human serum and running them 5 times on one plate, which was repeated 3 times on 3 nonconsecutive days. The LLOQ for the O1v2 OPA was 11, the O2a OPA was 8, and O2afg SBA was 12 (Table 3).

### OPA and SBA cross-reactivity and specificity.

**FIG 5:**
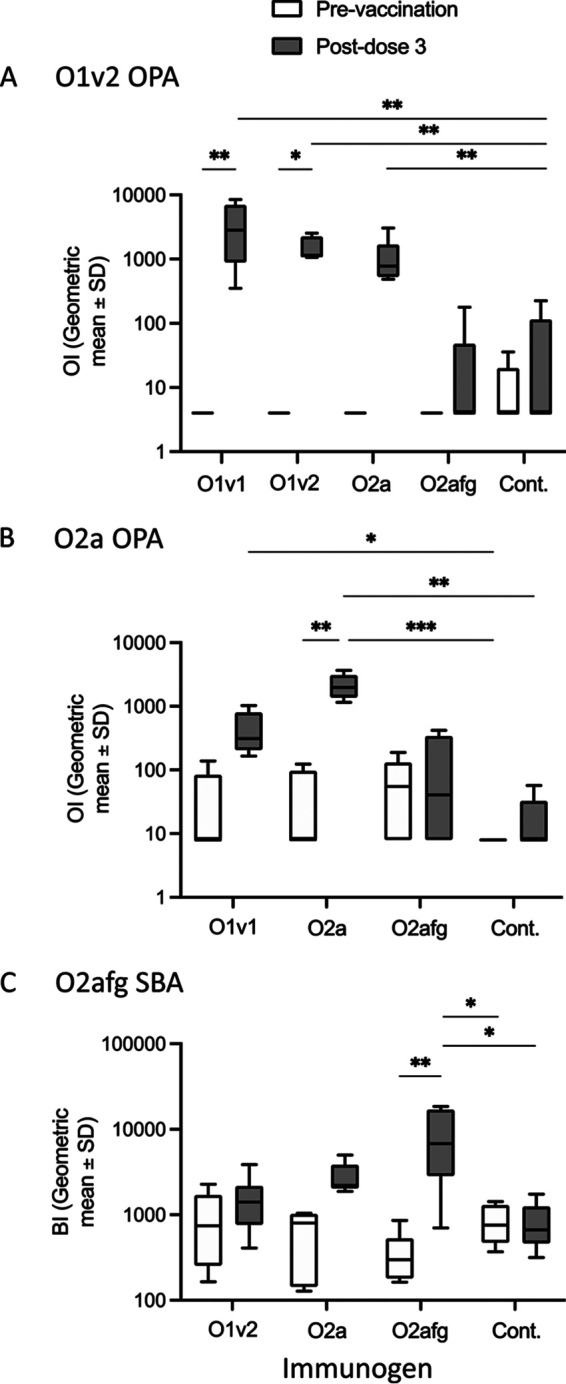
Antibody function measured by OPA or SBA. Rabbit sera from before (prevaccination) and after (postdose 3) vaccination with monovalent bioconjugate vaccines against O1v1, O1v2, O2a, and O2afg (*n* = 6 per group) or control (PBS) were run on the O1v2 K136 OPA (A), O2a K18 OPA (B), and O2afg K140 SBA (C). The opsonic index (OI) or bactericidal index (BI) was determined for each. Comparisons between paired pre- and postvaccination time points were made using the Wilcoxon signed-rank test and between vaccination groups using Kruskal-Wallis test with Dunn’s correction for multiple comparisons. *, *P < *0.05; **, *P < *0.01; ***, *P < *0.001.

Homologous and heterologous killing were tested using serum from rabbits vaccinated with monovalent bioconjugate vaccines. O1v2 K136 was killed with homologous anti-O1v2 serum and heterologous anti-O1v1 serum with nonsignificant killing induced by anti-O2a serum (Fig. 5A). In contrast, killing of O2a K18 and O2afg K140 was induced with only homologous anti-O2a/anti-O2afg serum (Fig. 5B and C).

The specificity of the assays was tested by preincubating monovalent rabbit serum with homologous and heterologous purified LPS. In the O1v2 OPA, killing by O1v2 antiserum was inhibited completely after preincubation with both O1v1 and O1v2 LPS but not O2a or O2afg LPS (Table 4). When the O2a OPA was performed, preincubation of O2a antiserum with O2a LPS and O1v2 LPS completely blocked killing, and O1v1 LPS partially inhibited killing. Preincubation with heterologous O2afg and O3b LPS resulted in no inhibition (Table 4). In the O2afg SBA, killing was completely blocked by preincubating O2afg antiserum with homologous O2afg LPS, and there was no inhibition in the presence of heterologous O1v1, O2a, and O3b LPS (Table 4); however, O1v2 LPS was able to partially inhibit killing in this assay (Table 4).

**TABLE 4:** Functional assay specificity

LPS/bioconjugate	Results by assay					
	O1v2 OPA^*a*^		O2a OPA^*b*^		O2afg SBA^*c*^	
	OI	Fold change vs zero inhibition control	OI	Fold change vs zero inhibition control	BI	Fold change vs zero inhibition control
None (zero inhibition)	43,443		3,190		2,827	
O1v1	<11	3,949.4	22	145	1,427	2.0
O1v2	<11	3,949.4	<8	398.8	354	8.0
O2a	50,251	0.9	<8	398.8	2,959	1.0
O2afg	64,426	0.7	2,613	1.2	<12	235.6
O3b			1,523	2.1	1,269	2.2

a Anti-O1v2 preincubation with LPS.

b Anti-O2a preincubation with LPS.

c Anti-O2afg preincubation with LPS.

## DISCUSSION

We have developed and optimized functional antibody assays for a number of clinically relevant K. pneumoniae strains and a 6-plex serological binding assay that allow for the measurement of anti-K. pneumoniae immunity after vaccination. These antibody function assays were adapted from the validated Streptococcus pneumoniae OPA which makes use of a baby rabbit complement as an exogenous source of complement and human promyelocytic leukemia cells as the phagocytic cell population (17). The use of batch-tested, quality-controlled reagents allows the qualification of such assays for use in vaccine studies requiring validated immunogenicity assays. Here, we assessed assay precision, specificity, and accuracy, which is an important step toward assay qualification to aid in K. pneumoniae vaccine development.

A multiplex antibody binding assay to measure antibody concentration after vaccination enables simultaneous measurement of antibodies against multiple strains expressing various O antigens. Compared with standard enzyme-linked immunosorbent assay (ELISA) techniques, this assay reduces the volume of serum required, the time taken to analyze, and the number of plates/reagents required to gain the same amount of data. These assays can be easily used in a cost-effective high-throughput manner in large-scale vaccine studies. Luminex technology has been used to develop serology assays for other bacterial infections, including the validated pneumococcal capsular polysaccharide assay (18, 19).

When the Luminex assay performance was assessed, variation was less than 15%, demonstrating acceptable levels of variation in a multicomponent immunological assay. We also show low %CV between replicates and in accuracy tests (less than 10%) and high specificity and linearity (R^2^, ≥0.90) across all serotypes tested, suggesting little modification of the antigenic regions of LPS in the bead conjugation process. Similar MFI found over two bead batches suggests good bead conjugation reproducibility; however, more work will be required to show reproducibility over a longer time frame and after increasing conjugation volumes for high-throughput testing. This assay can be adapted for human serum by altering the detection anybody and can be used to measure IgM or IgA antibodies and perhaps also antibodies in saliva or nasal wash after infection (20).

Functional assays to measure antibody-induced killing of three different strains of K. pneumoniae were optimized and qualified. The strains used contribute to the majority of human infections; O1, O2, and O3 serotypes and subtypes made up 88.9% of isolates in a recent report (5), and the K-types used in the study were human-disease-causing clinical isolates. Intra-assay variation was less than 10% across all serotypes, ranging from 4 to 14%; interassay variation was higher at 21.1% across all serotypes and was less than 30% across all serotypes. This level of variation is comparable to those observed for the Streptococcus pneumoniae OPA (21–23). The assay was specific as homologous killing was completely inhibited by homologous LPS and there was no inhibition with heterologous (non-cross-reactive) LPS (less than 3-fold change in OI/BI). Other measures of assay performance were low; %CV in accuracy tests was below 20%, and the linearity results were close to 1.0 (R^2^ = 0.947 across all serotypes), again aligning with results of other studies.

The O1 and O2 serotypes of K. pneumoniae share d-galactan domains, whereas the O3 serotypes share mannose domains. In qualifying these assays, we saw a strong cross-reactivity between the O1 and O2 serotypes. High cross-reactivity between O1v1 and O1v2 due to the shared d-galactan II with no difference in binding and function between the two suggests that antibodies against them can be used interchangeably. Cross-reactivity between O2a and O1v1 strains due to shared d-galactan I and between O1v2 and O2afg due to the shared d-galactan III antigen was also measured. Strong cross-reactivity between O1 serotypes but lower reactivity of O1v1 toward O2a or O1v2 toward O2afg indicates an immunogenic preference of the d-galactan-II antigen in O1 serotypes.

Interestingly, the anti-O2a serum was able to bind O1v2 LPS despite no shared antigen, but not vice versa, suggesting a debranching of d-galactan III to d-galactan I of the O1v2 LPS. This idea was confirmed by O1v2 LPS partially inhibiting antibody binding to O2a LPS (27% inhibition) and fully inhibiting killing of O2a K18 in response to anti-O2a sera in the O2a OPA. Furthermore, O2a LPS was unable to inhibit homologous killing of O2afg K140 in response to anti-O2afg serum in the SBA, confirming that the killing in the SBA was dependent on d-galactan III. Similar to other studies, there was limited cross-reactivity between O3 and O3b, weak binding, and only partial inhibition by the opposing LPS in specificity tests (24). Development of further functional assays against more serotypes will help elucidate the cross-reactivity between K. pneumoniae antibodies generated by vaccination and the potential for cross-reactivity to lead to vaccine-induced protection. K. pneumoniae was reported to cross-react with the pneumococcal type 19F polysaccharide (25), and thus, a K. pneumoniae vaccine may provide further beneficial effects as MenB-4C provides some protection against Neisseria gonorrhoeae (26).

In summary, we have developed and optimized binding and functional assays to measure anti-K. pneumoniae immune responses after vaccination. The standardization of assays such as these is important for the ongoing development of vaccines against this critical infection.

## MATERIALS AND METHODS

### Ethical consideration for animal care.

All of the experimentation involving animals was done under the frame of ethical protocol CE/Sante/E/001 (immunization and production of sera/polyclonal antibodies) approved by the ethical committee of CER Groupe (agreement number LA1800104). Agreement LA1800104 was bestowed by the Federal Public Service of the Walloon Region (Belgium). The experimentation was performed according to legislation in force at the moment of the studies, thus following the guidelines established at the European level (Directive 2010/63/EU revising Directive 86/609/EEC on the protection of animals used for scientific purposes), the Belgian level (Arrêté Royal Relatif à la Protection des Animaux d’Expérience, AR 2013/05/29), and the regional level (Code Wallon du Bien-Être Animal 03/10/2018).

CER Groupe is compliant with all regulations and guidelines for the care, welfare, and ethical treatment of animals and, as a minimum, with the following core principles: access to species-appropriate food and water; access to species-specific housing, including species-appropriate temperature and humidity levels; access to humane care and a program of veterinary care; the ability to demonstrate species-specific behavior; adherence to Replacement, Reduction and Refinement (3R) principles in the design of *in vivo* studies; study design reviewed by an institutional ethical review panel; commitment to minimizing pain and distress during *in vivo* studies; and work performed by appropriately trained staff.

### Rabbit immunization and sera.

New Zealand White rabbits, 3 to 4 months old, were vaccinated intramuscularly three times at 2-week intervals (day 0, 14, and 28) with monovalent anti-K. pneumoniae O-antigen bioconjugate vaccines or a phosphate-buffered saline (PBS) control. Bioconjugates containing O-antigen and EPA carrier protein (exotoxin protein A of Pseudomonas aeruginosa) were produced *in vivo* in E. coli, purified, and formulated in PBS buffer (KpO1v1-EPA, KpO1v2-EPA, KpO2a-EPA, KpO2afg-EPA, KpO3-EPA, and KpO3b-EPA) (27–29). Each vaccine contained a 1-μg polysaccharide (PS)-serotype dose in 0.5 mL. Serum was collected prior to immunization (prevaccination), 2 weeks after the second injection (postdose 2, day 28) and 2 weeks after the third injection (postdose 3, day 42). IVIG was also used as a positive control (Hizentra, 200 mg/mL).

### Bacterial strains.

For LPS purification, capsule mutants were generated at LimmaTech Biologics (LMTB) using well-characterized strains available in public strain collections. O1v1 strain NCTC 11682 (National Collection of Type Cultures), O1v2 NCTC 9127, O2a NCTC 9163, and O3b NCTC 13439 were purchased from Public Health England (PHE). O2afg NCTC 9147 was purchased from Polish Collection of Microorganisms (PCM), and O3 NCTC 9178 was purchased from Staten Serum Institute (SSI). Functional assays were performed using clinical isolates obtained by LMTB (Kp52_S16: O1v2_KL136 and Kp19_S12: O2afg_KL140) or the Microbiology Laboratory at Great Ormond Street Children’s Hospital NHS Trust (University College London [UCL] no. 1: O2a_KL18).

### LPS purification.

LPS was extracted by a combination of phenol water extraction as described previously (30) and purification using size exclusion chromatography to eliminate peptides, nucleotides, and other impurities. Briefly, bacterial cells were lysed in sodium dodecyl sulfate (SDS; Sigma-Aldrich) buffer, and proteins were removed by proteinase K digestion (Sigma-Aldrich). SDS was removed by ethanol precipitation (100% ethanol, ice-cold), and RNA and DNA were eliminated by RNase and DNase treatment (DNase I from bovine pancreas and RNase A from bovine pancreas; Sigma-Aldrich). Finally, the LPS was isolated by hot phenol-water extraction from the crude extract and further purified by size exclusion chromatography (Sepharose CL-6B column; Sigma-Aldrich) in the presence of SDS. The identity of LPS was confirmed by nuclear magnetic resonance (NMR; data not shown).

### Multiplex Luminex serological assay.

Magnetic beads were coupled to K. pneumoniae LPS as follows. LPS was added to 0.01% NaOH 0.0001% phenolphthalein (PPT) and vortexed, cyanuric chloride was diluted to 50 mg/mL and added to a final concentration of 0.5 mg/mL, and the solution was vortexed until colorless. Poly-l-lysine hydrobromide (PLL) (Sigma-Aldrich) was added to a final concentration of 5 μg per mg of LPS, and the solution was incubated at 4°C overnight. The solution was passed through a Sephadex G-25 column. MagPlex magnetic microspheres (Bio-Rad) were vortexed and sonicated, washed in 50 mM HEPES buffer, and pelleted using a DynaMag-2 magnetic particle concentrator (Invitrogen). Beads were activated with 5 mg/mL Sulpho-NHS and 5 mg/mL EDC (Thermo Fisher) on a Rotamix system for 20 min at room temperature. Beads were washed, and LPS-PLL was added in 50 mM HEPES buffer at various concentrations, vortexed, and placed on a Rotamix instrument for 120 min at room temperature. Beads were counted using a hemocytometer, resuspended at 1 × 10^7^/mL, and stored at 4°C in PBS containing 0.1% bovine serum album (BSA) and 0.05% sodium azide, which was protected from light.

The Luminex assay was carried out as follows. LPS coupled beads were diluted and combined in 6-plex (2,000 per serotype per well) in PBS containing 1% BSA and 0.05% Tween 20 (assay buffer). Beads were incubated with diluted individual monovalent sera, multivalent standard sera (a pool of postdose 3 immunization sera from each monovalent vaccinated rabbit), or multivalent quality-control (QC) sera (pooled postdose 2 sera) (assay buffer alone was used for the blank wells). The plate was sealed and incubated with shaking at 500 rpm at room temperature for 1 h. Plates were washed two times using a Bio-Rad Bio-Plex pro wash station. A R-phycoerythrin conjugated goat anti-rabbit IgG detection antibody (Sigma) at 3 μg/mL was added to each well and sealed and incubated with shaking at 500 rpm at room temperature for 30 min. Plates were washed three times, beads were resuspended in assay buffer, and plates were analyzed on a Luminex 200 system (Luminex Corp.) on the high photomultiplier tube (PMT) setting.

Luminex MFI data were converted to arbitrary unit (AU) data by interpolation from the standard curve generated for each serotype; the top dilution of the multivalent standard serum was assigned an AU of 1,000 for calculation. Qualification was performed using pooled multivalent QC serum. Intra-assay precision was determined from AU data obtained from 8 replicates (run in duplicate) on 1 plate, and interassay precision was determined from 3 separate repeats on 3 nonconsecutive days. Linearity was assessed by running nonimmune serum (antibody-depleted human serum; Pel-Freez) spiked with 4 different dilutions of QC sera; the correlation between initial sample dilution and resulting AU was assessed.

### Bacterial growth.

Working stocks of K. pneumoniae were generated by streaking a fleck of frozen master stock onto horse blood agar (Fisher Scientific) and incubating overnight at 37°C and 5% CO_2_. Todd-Hewitt Broth (THB) (Sigma-Aldrich) was inoculated with a single colony and grown at 37°C and 5% CO_2_ to an optical density at 600 nm (OD_600_) of 0.6 to 0.7, which was considered to be the late-log phase of growth. Once the required OD_600_ was reached, cultures were harvested and stored at −80°C in tryptone soy broth (Fisher Scientific), 0.5% d-glucose (Sigma-Aldrich), and 10% glycerol (Sigma-Aldrich) until required. The optimal dilution of bacteria for use in the assay was predetermined by spotting 10 μL of serially diluted bacteria onto LB (Lennox) (Sigma-Aldrich) agar to obtain a CFU count of 50 to 200 in an active complement, no serum control.

### HL-60 cells.

Human promyelocytic leukemia-60 (HL-60) cells (ATCC Standards) were differentiated into neutrophil-like cells by stimulation with 0.8% N,N-dimethylformamide (DMF) (Sigma-Aldrich) in RPMI 1640 (Invitrogen) supplemented with 10% fetal calf serum (FCS; HyClone) plus 1% l-glutamine (Invitrogen) for 5 to 6 days. The neutrophil-like phenotype of DMF-differentiated HL-60 cells was assessed prior to use by flow cytometry; a 55% increase in expression of CD35 and a 15% decrease in the expression of CD71 were considered acceptable for use in the assay (31). Fluorescently labeled antibodies used for flow cytometry staining were as follows: mouse anti-human CD35 FITC (Serotec) and mouse anti-human CD71 PE (Becton Dickenson).

### Opsonophagocytic killing assay.

The OPA was adapted from the validated Streptococcus pneumoniae OPA (17). In brief, serum samples were heat inactivated at 56°C for 30 min and serially diluted in opsonization (OPS) buffer (Hanks balanced sale solution [HBSS; +Ca/Mg] containing 10% FBS plus 1% gelatin solution), in a 96-well round-bottom plate. Working stocks of bacteria were thawed, washed, and diluted to the predetermined optimal dilution in OPS buffer and added to the serum; plates were incubated at room temperature on an orbital shaker at 700 rpm for 30 min. Baby rabbit complement (BRC) (Pel-Freez) at the predetermined optimal dilution (12.5% final) and differentiated HL-60 cells at a concentration of 1 × 10^7^/mL were then added to each well and incubated at 37°C and 5% CO_2_ on an orbital shaker at 700 rpm for 45 min. Two complement controls were included (a heat-inactivated [control A] and an active complement control [control B], with both containing bacteria and cells with no serum) to determine the level of nonspecific killing (NSK) in the assay.

Plates were placed on ice for 20 min, and 10 μL of the reaction mixture was spotted onto LB agar (1.5% agar) and allowed to dry. LB overlay agar (0.75% agar) was then poured over each plate, and inverted plates were incubated at 37°C and 5% CO_2_ for 16 to 18 h. The number of CFU were enumerated using an automated colony counter and ProtoCOL3 software (Synbiosis).

### Serum bactericidal assay.

SBA was developed based on the high-throughput *Shigella* serum bactericidal assay with several modifications (32). Serum samples were heat inactivated and serially diluted as above, and bacteria were thawed, washed, diluted to the predetermined optimal dilution, and added to the plate. BRC was added to each well, and the two complement controls (controls A and B) were used as above with the absence of HL-60 cells. Plates were incubated at 37°C and 5% CO_2_ on an orbital shaker at 500 rpm for 1 h. Plates were placed on ice for 20 min; 10 μL of the reaction mixture was spotted onto LB agar as above, LB overlay agar was added, and plates were incubated at 37°C and 5% CO_2_ for 16 to 18 h. CFU were enumerated as above.

### Statistics.

The OI/BI of a sample was calculated as the dilution of serum that kills 50% of bacteria using Opsotiter software (Bacterial Respiratory Pathogen Reference Laboratory, University of Alabama at Birmingham, USA). The percentage of nonspecific killing in each assay was calculated as 1 − (CFU[control B]/CFU[control A]) × 100, with an acceptability criterion of <35% applied to each assay plate. Graphing and statistical analysis were performed using Prism version 7.01 (GraphPad). Comparisons between paired pre- and postvaccination time points were made using the Wilcoxon signed-rank test and between vaccination groups using the Kruskal-Wallis test with Dunn’s correction for multiple comparisons.
