# Supplementary figures and images for: The Development of Immunological Assays to Evaluate the Level and Function of Antibodies Induced by Klebsiella pneumoniae O-Antigen Vaccines
Source: mSphere. 2023 Mar 6;8(2):e00680-22. doi: 10.1128/msphere.00680-22 (PMC10117086; doi:10.1128/msphere.00680-22)

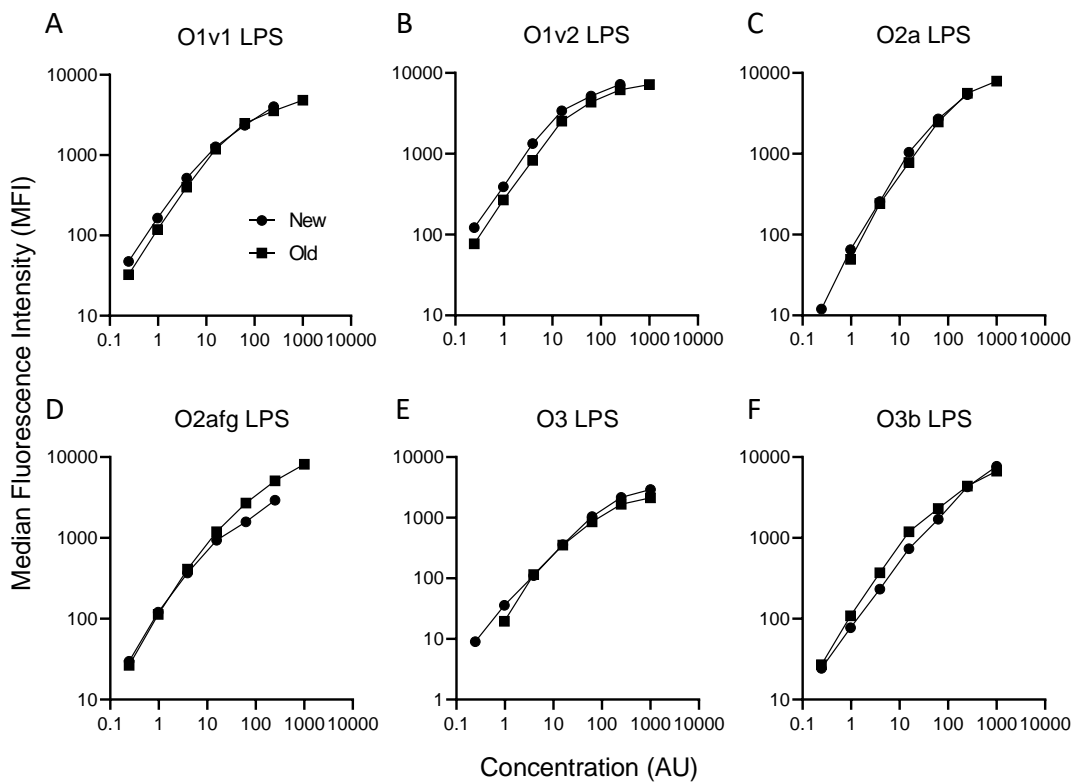

Supplement: FIG S1 [file msphere.00680-22-s0001.pdf]
